# Treatment of Delirium Tremens and of Acute Delirium

**Published:** 1881-03

**Authors:** 


### TREATMENT OF DELIRIUM TREMENS AND OF ACUTE DELIRIUM.

Dr. Rousseau treats ordinary cases of delirium tremens with large and repeated doses of bromide of potassium, which he thinks succeeds better than opium, especially when given in an early stage (*Annales Médicopsychologiques*). Only once has he met with the grave form assuming the febrile and delirious character. Remembering the treatment of Dr. Féréol in a similar case, he did not hesitate to adopt his method. After all the usual remedies had been exhausted without benefit, the physician in charge recommended the removal of the patient to a lunatic asylum. On admission he was extremely violent, flushed, eyes glistening, temperature elevated, pulse quick, full, and regular. He was incoherent, delivered interminable monologues, his attention could not be secured. He maintained that he had killed his wife and father, and expected every moment to be arrested by the police. He had hallucinations, and heard voices which made the most singular proposals to him. He thought himself in a forest, and took the persons around him for robbers. He committed a thousand extravagances, executed feats of strength, could not bear his clothes, sought a river to drown himself. From time to time he fell into a stupor with carphology, tried to seize small objects on his clothes, then suddenly awoke. There was a general tremor, speech was embarrassed, the tongue furred, the pupils contracted, and but little sensitive to light, the left a little more dilated than the right, the skin was anæsthetic, the head not apparently painful.

Ten grammes of potassium bromide were administered on admission, but the agitation and delirium continued during the day and night. The bromide was continued, and on the following day he kept for seven hours in a *cool* bath (*bain frais*), cold compresses being applied to his forehead from time to time. Whilst in the bath he became calm, with a marked diminution of the delirium and trembling. He passed an excellent night and made a rapid recovery, but continued as a precautionary measure to use the bromide, taking altogether fifty grammes in five days.

The same treatment was also adopted with success in a non-alcoholic case of acute delirium with extreme excitement—"one of those congestive manias which form the substratum of general paralysis." There was slight embarrassment of speech, pupils unequally dilated, pulse strong and quick, skin burning, the face red and congested. He cried, danced, committed all sorts of extravagances, refused to lie down, stripped himself naked, clasped the coverlet between his arms, saying he was going to be confined of a reptile. He was completely delirious. Ten grammes of bromide of potassium produced no diminution of excitement during the night after admission. On the following day a cool bath was administered for seven hours with repeated cold irrigations of the head, the temperature of which was excessive. The agitation subsided, and intelligence was partially restored during the bath, after which tranquility was uninterrupted. Mental confusion and delusions still remaining, he was purged for five days by calomel and aloes, at the end of which time he was perfectly sane, with speech and pupils normal. Seen from time to time since, he continues quite well, now nine months since the attack.—*Medical Press and Circular.*

